# Iron Status in Sport Horses: Is It Important for Equine Athletes?

**DOI:** 10.3390/ijms26125653

**Published:** 2025-06-12

**Authors:** Paula Kiełbik, Olga Witkowska-Piłaszewicz

**Affiliations:** Department of Large Animal Diseases and Clinic, Institute of Veterinary Medicine, Warsaw University of Life Sciences, 02-787 Warsaw, Poland

**Keywords:** iron deficiency, equine athlete, sport horse, hepcidin, iron metabolism

## Abstract

Iron is unquestionably an essential element of physical performance for horses, just as it is for many other animals, including humans. Although post-exercise equine iron deficiency is not a common problem, recent studies showed that equine athletes may be considered a model for human exercise physiology. Sports anemia among human athletes is a common nutritional issue and remains one reason for poor physical fitness. Thus, this study area needs comprehensive knowledge since iron homeostasis changes in equine athletes remain unrecognized. The current review aims to summarize studies describing iron metabolism changes in response to physical effort in equine sports medicine. The confirmed prevalence of gastrointestinal bleeding, hemolysis, and hematuria in horse athletes seems to play a role in iron metabolism. Similarly, exercise-induced inflammation and its effect on the iron key regulator in mammals—hepcidin—may be as crucial for overall iron homeostasis in horses as in humans. In this review, we also present available data regarding the possible effect of various hormones on iron metabolism, performance-enhancing strategies related to iron metabolism in horse athletes, and the clinical relevance of regular iron status monitoring in sport horses. Overall, this article aims to discuss current knowledge and highlight existing gaps in our understanding of iron homeostasis in sport horses.

## 1. Introduction

Iron is an essential mineral for horses and many other animals. It plays key roles in physiological processes—as a component of hemoglobin and myoglobin for oxygen transport, a cofactor for metabolic enzymes, and a regulator of immune function and development [1,2]. Iron is crucial for physical performance. In human athletes, iron deficiency (sports anemia) is common, affecting up to 35% of females and 11% of males [3,4,5]. Notably, athletes often show reduced hemoglobin levels due to expanded plasma volume, a phenomenon known as hemodilution [6]. Iron loss may stem from both physiological demands and pathological conditions like gastrointestinal bleeding and inflammation. Therefore, dietary iron monitoring is essential during training, with baseline tests including hematocrit (Hct), hemoglobin (Hb), red blood cell (RBC) count, and serum ferritin [7,8].

In sport horses, iron is similarly important, though post-exercise deficiency is uncommon [9,10]. Still, horses have been proposed as models for human exercise physiology and musculoskeletal disorders, including osteoarthritis and tendon injuries [11,12,13], making them potentially relevant in sports anemia studies.

This review examines how exercise affects iron metabolism in horses, comparing findings to human data. It discusses mechanisms of iron loss in horses, such as bleeding, hemolysis, and hematuria, and highlights that intense or prolonged exercise may trigger inflammation, influencing iron status similarly to humans. The review also explores the effects of physical effort on hepcidin, erythropoiesis, inflammatory cytokines (e.g., IL-6), and overall iron balance. While such mechanisms are well-documented in humans, equine data remain sparse and often inconsistent. Furthermore, iron status monitoring tools and hematological markers used in sport horses are discussed, emphasizing the need for more research to clarify equine iron metabolism.

## 2. Iron Homeostasis

Understanding the mechanisms of iron absorption and distribution is essential in the context of iron deficiency. These processes involve specific receptors, transporters, and regulatory proteins located mainly in the gastrointestinal tract, liver, and blood. Iron absorption occurs mainly in the first part of the small intestine (duodenum).

Iron is absorbed mostly in the duodenum, the first part of the small intestine. There, special cells called enterocytes take in iron in two main forms: heme iron (from animal foods) and non-heme iron (from plants). Heme iron is easier for the body to absorb but can be more damaging to cells. Non-heme iron is absorbed less easily but has well-known ways to enter the cells. On the surface of these cells, non-heme iron (which is in a form called Fe^3+^) is changed into a different form (Fe^2+^) by an enzyme—duodenal cytochrome b (Dcytb)—then transported into the cytoplasm via divalent metal transporter 1 (DMT1).

Once inside the enterocyte, Fe^2+^ may be stored or transported and released into the blood via ferroportin (Fpn), with the aid of ferroxidase hephaestin, which reoxidizes iron to Fe^3+^. In circulation, Fe^3+^ binds to transferrin (Tf) and is delivered to cells through transferrin receptor 1 (TfR1), present in most cell types. Transferrin receptor 2 (TfR2), expressed in hepatocytes, duodenal crypts, and erythroid cells, plays a regulatory role in iron homeostasis. In renal tubular cells, cubilin mediates transferrin-bound iron uptake [14,15,16]. The regulation of iron transport relies on controlled expression of these proteins, ensuring efficient uptake and distribution.

Hepcidin, a liver-derived hormone, is the central regulator of systemic iron balance. Elevated hepcidin degrades ferroportin, reducing iron efflux from enterocytes and macrophages. It blocks iron from leaving these cells, lowering iron availability [17,18]. A schematic summary of these pathways is provided in Table 1.

## 3. Iron and Sport Horses

**Figure 1:**
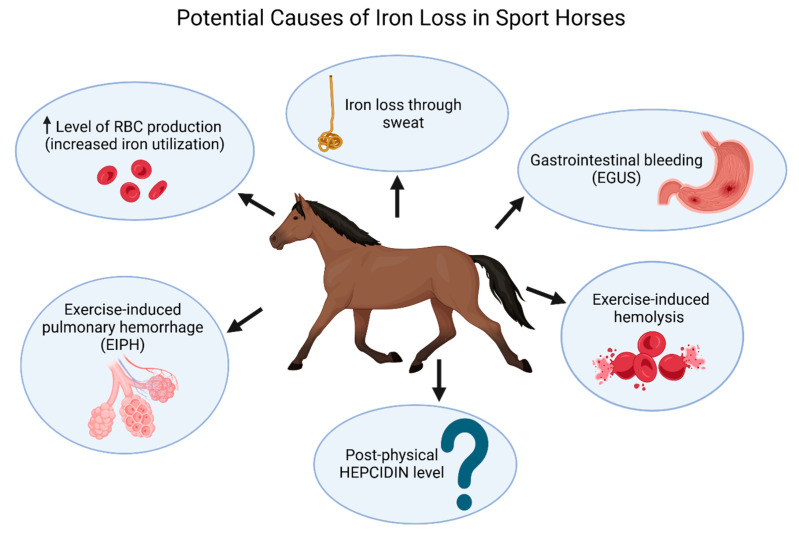
Schematic illustration of potential causes of iron loss in sport horses. Exercise-induced hemolysis, increased prevalence of gastrointestinal bleeding, and pulmonary hemorrhage (EIPH) have been confirmed as consequences of intense and/or prolonged training in equine athletes. All of these conditions may lead to blood and iron loss. Additionally, iron loss through sweat may also negatively affect the iron status of horses. Likewise, since hepcidin production increases in response to inflammation, exercise-induced chronic inflammation may potentially lead to hypoferremia.

In horses, approximately 85% of daily iron needs are met through erythrocyte recycling by macrophages, while 15% comes from gastrointestinal absorption [19]. These proportions may shift in horses undergoing regular training, though the effects of exercise on equine iron metabolism are not yet well-studied. Nevertheless, potential mechanisms of iron loss in sport horses are outlined below and in Figure 1. Like human athletes, sport horses face increased physiological demands affecting iron status. Intense exercise raises iron utilization due to elevated red blood cell production, essential for oxygen transport during physical activity. Additionally, prolonged and intense effort may lead to iron loss through sweat, as shown in humans [20,21]. However, in horses, sweat-related iron loss is generally considered minor. Equine sweat contains about 21 mg/L of iron, and although horses can lose 25–30 L of sweat daily during intense activity—equating to roughly 500 mg of iron—this is still seen as a relatively modest contribution to overall iron loss [9].

Exercise-induced destruction of red blood cells—hemolysis—is well-documented in human endurance training as a contributor to sports anemia. It results from repeated muscle contractions and vascular compression, leading to red blood cell membrane damage and hemoglobin release, which may cause toxic effects in surrounding tissues [22]. Similar effects have been observed in horses, specifically in endurance horses [23], racehorses [24,25], and during treadmill training [9,26]. In one study of 47 endurance Arabians, hemolysis markers were highest in horses eliminated for metabolic reasons compared to finishers and those withdrawn for lameness [23].

Gastrointestinal bleeding and ulcers are also linked to intense training in both human [27,28,29] and equine athletes [30,31]. Equine Gastric Ulcer Syndrome (EGUS) is especially common in racehorses, with prevalence reaching up to 95% [32,33]. High-intensity training, stress, limited forage, and travel contribute to ulcer development. Prolonged exercise diverts blood from the gastrointestinal tract to muscles and skin, impairing gut integrity and oxygenation. This may lead to lesions, bleeding, and potential iron loss, although the extent of net iron depletion remains uncertain.

Exercise-induced pulmonary hemorrhage (EIPH) is bleeding within the lungs during or after intense exertion [34]. Its pathophysiology is not fully known, but likely involves mechanical, hemodynamic, and inflammatory components, including pulmonary hypertension. While mild EIPH may not significantly affect iron concentrations, some studies suggest iron deficiency could play a role in pulmonary hypertension pathogenesis, and iron supplementation has shown benefits in rat models [35].

## 4. Post-Physical Hepcidin Level, Iron Status, and Inflammation in Sport Horses

**Figure 2:**
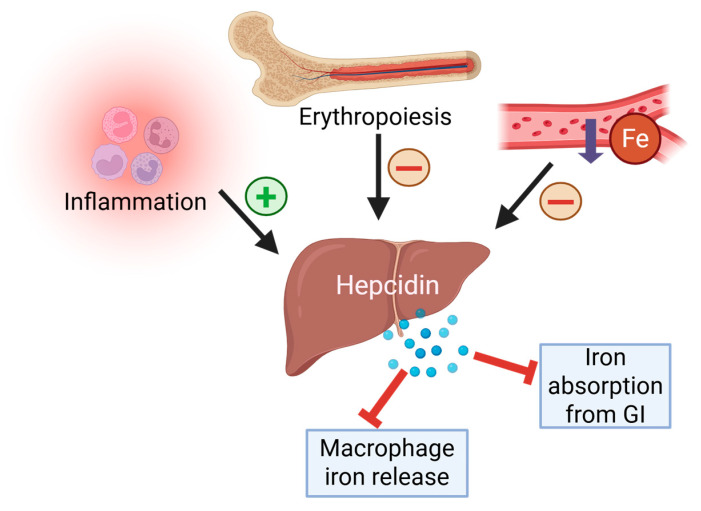
Factors influencing hepcidin levels and their role in iron regulation. Hepcidin expression is downregulated in response to iron deficiency and increased erythropoietic demand. In contrast, inflammation upregulates hepcidin as a host defense strategy, blocking iron absorption from the gastrointestinal (GI) tract and inhibiting iron release from macrophages.

The mechanisms of exercise-induced iron loss also include less direct processes related to hormone activity (mostly hepcidin) and induce an inflammatory response [36]. Hepcidin is a key hormone responsible for overall control of the iron status in the organism by inhibiting intestinal absorption of iron and iron release from organism stores (mostly within hepatocytes and macrophages). In humans, during iron deficiency, the hepcidin production by hepatocytes remains low, which increases iron concentrations. This feedback system controls the iron pool within the organism to maintain iron homeostasis. However, iron is not the only factor affecting the hepcidin level in the organism. Naturally, increased erythropoietic activity (red blood cell production) inhibits hepcidin production since this process is the predominant consumer of iron, because the body’s iron stores need to stay available, and high hepcidin levels stop that. Likewise, hepcidin production is raised in inflammation and infection as a host defense strategy to reduce iron available to pathogens [16,17,37]. Figure 2 visualizes the interplay between iron status, erythropoiesis, and inflammatory signaling in the regulation of hepcidin, highlighting its central role in maintaining systemic iron balance.

**Figure 3:**
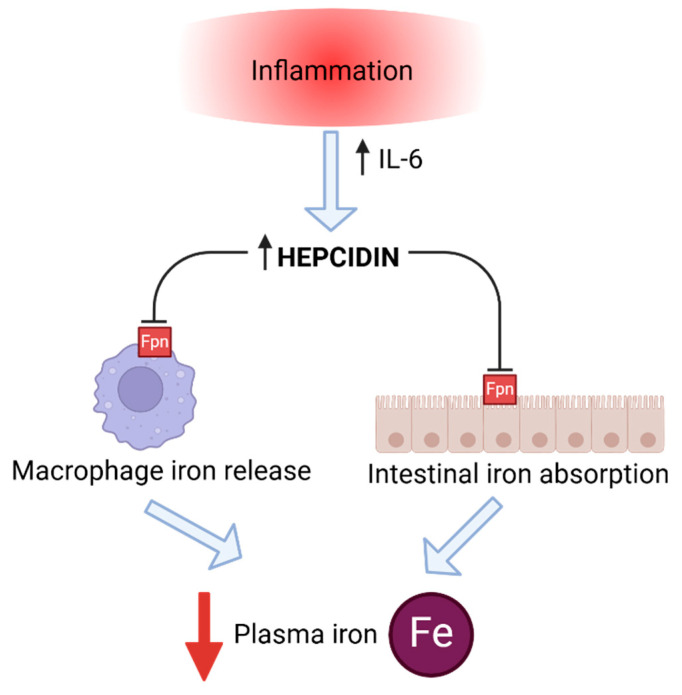
Effect of chronic inflammation (e.g., post-exercise inflammation) on hepcidin expression. Inflammatory cytokines (mostly interleukin-6) increase hepcidin levels, even in non-iron deficient individuals. Iron absorption and its release from stores may be progressively inhibited, potentially leading to a reduction in serum iron concentrations.

Recently, identifying the exact effect of physical exercise on iron metabolism has become one of the most important aspects in sports anemia-related studies. This also refers to hepcidin, especially since the post-exercise inflammation processes seem to influence hepcidin significantly, and the chronic increase of this hormone leads to iron deficiency (Figure 3). In human athlete studies, there seems to be a consensus on this issue—endurance exercise upregulates hepcidin levels [38,39,40,41,42]. According to the latest findings, lactic acid produced during anaerobic exercise plays a key role in regulating iron metabolism by modulating hepcidin expression [43]. During high-intensity exercise, increased lactic acid accumulation stimulates hepcidin production, resulting in reduced iron availability. Nevertheless, researchers note that an athlete’s baseline (pre-exercise) iron stores seem to play an essential role in the quantity of hepcidin response following endurance activity. Primary decreased iron status probably prevents the increase in hepcidin levels, even in the presence of an inflammatory response to the exercise. Similar studies were not conducted as extensively on horse athletes, but a few focused on exercise-induced inflammation and its effect on hepcidin concentrations.

An intense physical effort results in a stress response within the organism and a further probability of muscle damage. This condition triggers defense reactions like those in infections. Exercise-induced acute phase response (EIAPR) is a physiologically normal and essential process required to repair damaged tissues and adapt to the physical stress of training. However, excessive or chronic inflammation may be harmful to the organism, leading to various health issues and performance decrements [44,45]. As mentioned above, inflammatory response induces elevated hepcidin levels in non-iron deficient individuals. Subsequent inhibition of iron absorption and its release from stores can occur, potentially leading to a drop in serum iron concentrations. Among many inflammatory cytokines, interleukin-6 (IL-6) significantly determines hepcidin expression during inflammation [46,47,48]. It is worth mentioning that the IL-6 response to strenuous exercise increases more than that of any other cytokine, and it acts as an anti-inflammatory myokine. However, the increase depends on exercise intensity, duration, the mass of muscle recruited, and the endurance capacity of the athlete [49,50]. Regarding horse athletes, there is still limited research specifically investigating the correlation between exercise-induced inflammatory response and hepcidin levels. In one of the studies on horses, researchers found that inflammation induced by bacterial components up-regulated hepatic hepcidin mRNA expression, which quickly reduced the amount of iron circulating in the bloodstream [51]. In horse athletes, the inflammatory response itself to different types of physical effort is well-documented, but it appears to depend on the type of physical activity. One of the studies on jumping horses revealed that this type of exercise also induces an inflammation-like response [52]. On the contrary, acute exercise in horses (short distance race) did not induce an acute phase reaction elicited by inflammatory stimuli [53]. Likewise, another study on sport horses did not show changes in crucial inflammatory cytokines (including IL-6) following training of different intensity [54].

Differences in the activation of the inflammatory response after exercise may result from the various types of physical effort examined in the cited studies, similar to what is observed in humans. However, it is important to note that each study involved different horse breeds, which may complicate the comparison of results. Additionally, the study by Colahan et al. (2002) [54] was conducted on a small sample size (five horses), which may also affect the reliability of its findings. Furthermore, variations in the inflammatory response observed in these studies may be influenced by the horses’ training levels. Well-trained horses tend to develop an anti-inflammatory environment, whereas untrained horses often exhibit a pro-inflammatory reaction after exercise. This suggests that the anti-inflammatory state seen in well-trained racehorses is an adaptive response to increased workload during physical activity [55].

In conclusion, the data obtained from horse athletes results in confirmation of observations made on human athletes—exercise-induced inflammation varies depending on various factors, including the type of physical activity, its intensity, duration, or horses’ training level. Since chronic inflammation may be deleterious for organisms and potentially lead to increased hepcidin expression, resulting in a reduced amount of iron circulating in the bloodstream, further studies investigating the effect of various exercises on iron metabolism in horses are needed. Moreover, new tools for early monitoring of chronic/ongoing inflammation in sport horses are necessary and highly desired to prevent injuries. Optimal training should be a primary goal for breeders and trainers, as it helps promote an anti-inflammatory state during different types of physical activity [49,55].

## 5. Hormones Influencing Hepcidin and Iron Status

One of the hormones that increases after intense exercise is prolactin, likely as part of the body’s stress and adaptive response [56,57,58]. The exact mechanisms by which prolactin influences hepcidin and iron metabolism remain unclear, especially in equine species. In humans, some studies showed decreased hepcidin levels in hyperprolactinemic patients, with levels increasing after prolactin-suppressing therapy—without changes in inflammation or iron status [59]. However, other studies found no such relationship [60]. Given that prolactin receptors are expressed in the liver (the site of hepcidin production) [61], it is possible that this hormone exerts indirect regulatory effects on hepcidin expression. Nevertheless, the role of prolactin in hepcidin regulation is unclear, and more research is needed to understand its impact on iron metabolism and hepcidin expression. In horses, prolactin levels also rise after exercise and emotional stress, but to date, the specific influence of prolactin on iron metabolism or hepcidin regulation in equines remains unexplored, highlighting a gap in current knowledge [62,63,64].

Sex hormones also modulate hepcidin expression. In humans, testosterone suppresses hepcidin, enhancing iron availability—confirmed across multiple studies [65,66,67,68]. Testosterone is thought to downregulate hepcidin via androgen receptor-mediated pathways, which may increase iron absorption and erythropoiesis. Though not directly studied in horses, stallions show better hematological and iron profiles than mares and geldings, suggesting a similar role [69,70]. Testosterone is also part of the anabolic index used to assess equine fitness [71,72], increasing post-race but decreasing in endurance horses regardless of fitness. This likely reflects the different exercise types: short, intense efforts boost testosterone, while prolonged endurance exercise suppresses it due to sustained stress [73].

Estrogens also affect iron regulation. Estradiol (E2) has been shown to reduce hepcidin expression in mice and humans, likely as compensation for menstrual iron loss [60,73,74]. In women, the hepcidin response to exercise is lower during the early follicular phase, promoting iron absorption post-menstruation [75]. In mares, similar hormonal patterns have been observed, where E2 dominance during pre- and ovulatory phases corresponds to hepcidin inhibition and improved iron status, suggesting conserved estrogenic regulation of iron metabolism across species. In contrast, high progesterone concentrations during the luteal phase have been shown to increase ferritin levels and reduce serum iron in mares [76]. This effect is possibly mediated through the activation of progesterone receptor pathways that stimulate hepcidin biosynthesis. A study on zebrafish demonstrated that progesterone receptor membrane component-1 (PGRMC1) plays a regulatory role in hepcidin production [77]. This hormonal regulation likely reflects an adaptive mechanism to optimize iron homeostasis across the estrous cycle in mares. Progesterone rises after ovulation, stimulating hepcidin to promote iron storage, conserve iron, and prepare for potential pregnancy. Estradiol rises in the follicular phase to prepare for ovulation, inhibiting hepcidin and increasing iron mobilization to meet metabolic demands [76].

Growth hormone (GH), similarly to prolactin, also rises after exercise [78,79,80] and has been shown to reduce hepcidin in humans—both in GH-treated individuals and in patients with acromegaly [81,82]. This is likely due to GH’s stimulation of erythropoiesis and potential direct suppression of hepatic hepcidin synthesis via growth factors [83]. Though less studied in horses, exercise-induced GH elevation was observed in stallions and geldings, with increases persisting post-exercise, possibly contributing to immune and metabolic adaptations [62,63]. These findings suggest a potential role for GH in modulating iron metabolism during and after exercise in horses, but targeted research is needed to elucidate these pathways.

In conclusion, exercise induces changes in hormones such as prolactin, testosterone, estradiol, progesterone, and growth hormone, which likely influence hepcidin regulation and iron metabolism in horses, though the mechanisms are not well understood. Future research should investigate how different types of exercise affect these hormones and iron metabolism, clarify prolactin’s role in equine iron regulation, and explore hormonal effects on iron status during reproductive phases under physical stress. Understanding these interactions could improve training and nutritional strategies for athletic horses.

## 6. Monitoring Iron Status

In human athletes, common profiles of iron status include red blood cell (RBC) count; hemoglobin (Hb) concentration; hematocrit (Hct); serum iron (Fe), ferritin (Ferr), and transferrin (T) concentrations; T saturation (TSAT); total iron-binding capacity (TIBC); and unsaturated iron-binding capacity (UIBC) [84]. Moreover, optimization of various factors influencing iron status in human athletes has been studied extensively since iron metabolism can change physical performance [85,86,87]. However, such tests are not routinely performed on horses. Most of the published data relates to pregnant mares [88]. The reference values for horses are not well-established for all iron metabolism parameters. There are some examples established at Cornell University College of Veterinary Medicine’s website [89] which gives reference values for iron of 95–217 µg/dL, TIBC 289–535 µg/dL, and transferrin % saturation 27–56%. However, such data are unique to horses. Below, studies related to iron homeostasis in sport horses are discussed. The reviewed data, along with the corresponding publication sources, are also presented in Table 2.

According to research, sport horses exhibit significantly higher serum iron concentrations than recreational horses, likely due to intensified erythropoiesis, as elevated iron correlated with higher RBC, hematocrit, and Hb values [90]. In one study, only one group of horses showed increased serum iron after exercise, possibly due to varying protocols—suggesting that the type and duration of exercise differently affect iron homeostasis [91]. However, as other iron markers were not assessed, the mechanisms behind these changes remain unclear. It is worth noting that in horses, a substantial portion of RBCs is stored in the spleen at rest. Therefore, comparing hematological values before and after exercise not only reflects the dynamics of iron metabolism but also reveals the mobilization of the splenic reserve, providing additional insight into the horse’s physiological response and erythrocyte availability under exertion. As a result, resting HCT, RBCs and Hb values measured during training periods may be decreased, potentially leading to a false diagnosis of anemia if not interpreted in the context of exercise-related physiological changes.

In another study, serum iron was evaluated as a potential biomarker to eliminate risk before endurance rides, but results indicated it was unreliable. Iron concentrations remained within reference ranges, likely due to the horses’ high training level and low disease susceptibility [92]. Physical effort also affects plasma ferritin concentrations; longer or more intense exercise causes greater increases. Despite its role as a marker of iron stores, ferritin should be interpreted cautiously in horses, as it is also an acute-phase protein elevated in response to inflammation from tissue damage [93].

Assenza et al. reported increased serum ferritin, iron, and transferrin concentrations during repeated show jumping events, suggesting that iron elevation may result from hemolysis, not just storage mobilization [94]. Similarly, three-day eventing horses showed post-exercise increases in erythrocyte and iron indices, likely from splenic contraction [95]. On the other hand, studies on the effect of high-intensity, short physical exercise (gallop) on iron hemostasis revealed significant decrease after the exercise. This decrease might result from the mobilization of iron ions during the acute phase inflammatory response triggered by cellular stress and tissue damage occurring during intense exercise.

Findings on markers like transferrin, TIBC, and UIBC are inconsistent. One study showed increased TIBC and transferrin saturation, with reduced serum iron 30 min post-exercise—indicating a heightened need for iron during recovery [96]. Piccione et al. also noted elevated ferritin, transferrin, TIBC, and UIBC concentrations over a three-day jumping competition [95]. An increase in transferrin, TIBC, and UIBC levels following exercise may be attributed the body’s increased demand for iron, e.g., to support erythropoiesis. Nevertheless, in an 80-day training program, TIBC and UIBC decreased until day 60, then rose but did not return to baseline by day 80 [97]. The decline may reflect adequate dietary iron intake matched to the horses’ physiological demands. Additionally, gradual adaptation to training workload may have allowed for more efficient iron utilization and storage.

Elevated iron and related proteins may also partly reflect exercise-induced hemoconcentration [95,98], though a human study argued that serum iron increases post-exercise are not solely due to reduced plasma volume [99]. Although no direct scientific studies have confirmed the impact of subclinical iron deficiency on performance parameters such as VLa4 and V200 (parameters routinely used to evaluate performance [100]) in Gallopers and Standardbred trotters, there is indirect evidence suggesting that iron deficiency may negatively affect aerobic performance by reducing hemoglobin concentrations and impairing mitochondrial function. Human studies have shown that iron deficiency leads to mitochondrial dysfunction, resulting in decreased ATP production and diminished muscle contractility [101]; while these findings are based on human data, the underlying physiological mechanisms may be similar in horses.

Available studies on iron parameters are sometimes contradictory, which is probably due to various types of exercises (mostly duration and intensity), horse breed, varying levels of training, age, differences in nutritional management, as well as also study protocols, and performed blood tests. Future research should focus on longitudinal, controlled studies using standardized exercise protocols and dietary conditions to comprehensively investigate the impact of physical effort on iron metabolism. Additionally, understanding hepcidin concentrations, in conjunction with other markers, can provide insights into the horse’s overall iron metabolism. It would be particularly important to include markers of inflammation, as the inflammatory response affects iron homeostasis and may be influenced by the horses’ training levels [55]. The recommendations for iron metabolism monitoring in horses have been presented in Table 3.

## 7. Performance-Enhancing Strategies Related to Iron Metabolism: Benefits and Risks

Frequent iron supplementation in high-performance horses poses a risk of iron overload, potentially leading to oxidative stress and immune dysregulation if not properly monitored. Although horses have regulatory mechanisms limiting intestinal iron absorption, excessive or prolonged intake—especially through supplements or iron-rich water—can overwhelm these defenses. A case series by Theelen et al. (2024) reported haemochromatosis and hepatopathy in 21 horses and one donkey following long-term consumption of iron-rich surface water [102]. These animals had transferrin saturation >80% and histological signs of hepatic iron accumulation and fibrosis. While an 8-week high-dose oral supplementation in ponies did not cause liver lesions, it did significantly elevate serum iron, ferritin, and liver iron content, indicating potential risk with chronic exposure [103].

Performance-enhancing strategies like erythropoietin (EPO) administration and altitude training are also closely tied to iron metabolism. Early studies suggested low-dose recombinant human EPO might modestly improve performance in Standardbred horses [104], but later research raised safety concerns. EPO increases red cell mass and blood viscosity, potentially causing thrombotic events. Adverse effects include immune-mediated anemia, thrombosis, hypertension, and cardiac strain. Horses may develop antibodies neutralizing both exogenous and endogenous EPO, leading to non-regenerative anemia and long-term complications, making EPO use both ineffective and hazardous [105].

Altitude training induces physiological adaptations such as greater blood volume and elevated 2,3-diphosphoglycerate (2,3-DPG), which may enhance aerobic capacity at sea level. Horses acclimatized for 9 days at 3800 m showed improved heart rate and lactate recovery post-exercise [106]. However, hypoxia may reduce training intensity and raise pulmonary artery pressure, increasing vascular risk. Although some studies report improved muscle metabolism and lactate clearance, results are inconsistent and based on small samples [107]. Assessing erythropoietic response in horses is further complicated by their large splenic red cell reservoir, which masks blood changes unless techniques like adrenaline administration or splenectomy are used—each with its own limitations. While hypoxic training is effective in humans, its role in horses remains uncertain and requires further standardized research.

## 8. Clinical Relevance

Regular monitoring of iron status in sport horses may support early identification of subclinical alterations that could impact performance or recovery. Although overt iron deficiency is considered rare in adult horses [9,108], certain physiological and environmental conditions—such as high-intensity training, exercise-induced hemolysis, gastric microbleeding, or inflammation—might influence iron metabolism and complicate the interpretation of routine hematological profiles [108,109,110,111,112].

Sport horses often exhibit physiologically elevated Hct, Hb, and RBC due to increased erythropoietic activity and splenic contraction during exertion, especially post-exercise values [113,114,115,116]. As a result, values falling within the lower end of the reference range (e.g., Hct 38–40%) might, in the context of high-performance athletes, reflect a relative decline in red cell mass rather than normal variation. Therefore, interpretation of hematological and iron-related biomarkers in equine athletes should always consider the training phase, time post-exercise, hydration status, and discipline-specific adaptations [113,114,115,116,117,118]. Post-exercise changes of some blood parameters in race horses across different breeds and race distances has been presented in Table 4. Additionally, during intense exercise, horses may experience exercise-induced hemodilution due to plasma volume expansion, which can transiently lower hematocrit and hemoglobin concentrations without affecting total red cell mass [117,118,119].

Iron supplementation should not be administered empirically [9,108]. According to current expert recommendations, including those by Dr. Eleanor Kellon, it is more detrimental to have excessive iron levels than insufficient ones [120]. Excess iron can lead to oxidative stress, damage to erythrocyte membranes, and secondary deficiencies of essential minerals such as copper, which impairs red blood cell production.

Over-supplementation may pose a risk, particularly in horses receiving injectable preparations or consuming water from iron-rich sources [102,121]. Although the literature on iron overload in horses is limited, isolated reports describe adverse outcomes such as hepatic damage and oxidative stress following excessive parenteral iron administration [102]. Given that injectable iron bypasses normal absorption regulation, it should be used only under veterinary supervision and after clear diagnostic confirmation.

In conclusion, while iron homeostasis in horses is generally well-maintained, the unique demands placed on sport horses may expose vulnerabilities that warrant closer observation. Further studies are needed to validate the clinical significance of subtle iron disturbances in this population. A cautious, evidence-informed approach to assessment and management is recommended [122]. A proposed summary of diagnostic considerations and clinical decision points is presented in Table 5.

## 9. Conclusions

Iron deficiency is rare in sport horses, so the exact impact of physical effort on iron homeostasis in equines remains unclear. Studies in humans show how iron metabolism changes in response to physical exertion in athletes and highlight its importance for achieving optimal athletic performance. Therefore, this area may also warrant investigation in horses, especially considering that multiple factors can influence iron status in equine athletes. Gastrointestinal bleeding, hemolysis, pulmonary hemorrhage, and hematuria have been observed in both human and equine athletes. Similarly, the effect of exercise-induced inflammation on hepcidin concentrations appears inconsistent in sport horses and likely depends on factors such as exercise type, intensity, duration, and baseline iron stores. Notably, the influence of hormones—especially sex and stress-related hormones—on hepcidin and iron metabolism remains one of the least explored areas in equine sports medicine. Iron status monitoring in horse athletes is rare, leaving gaps in our understanding of how physical activity affects iron balance. Iron homeostasis in sport horses is a complex and dynamic process influenced by multiple factors including exercise type, intensity, duration, breed, training level, age, and nutritional management. Current evidence suggests that monitoring iron parameters alongside inflammatory markers and hepcidin concentrations could provide a more comprehensive understanding of iron metabolism in sport horses. The discussed performance-enhancing strategies targeting iron metabolism involve notable risks and demonstrate inconsistent benefits in horses. Without careful monitoring, they may lead to adverse effects, thus further research is needed to assess their safety and efficacy. Moreover, regular monitoring of iron-related markers can aid in the early detection of imbalances, and individualized management is key to supporting both performance and long-term health.

## Figures and Tables

**Table 1:** Iron metabolism pathways: absorption and distribution.

Process	Key Components	Iron Form	Function
Iron Absorption [14]	Duodenum, Enterocytes	Fe^2+^ (heme) or Fe^3+^ (non-heme)	Absorption of iron from food
Reduction of Non-Heme Iron [15,16]	Duodenal cytochrome b (Dcytb)	Fe^3+^ → Fe^2+^	Reduction of non-heme iron to assimilable form
Iron Transport into Enterocytes [14,15,16]	Divalent metal transporter 1 (DMT1)	Fe^2+^	Transport of Fe^2+^ into enterocyte cytoplasm
Iron Utilization or Export [14,15,16]	Ferroportin (Fpn), Hephaestin	Fe^2+^ → Fe^3+^	Internal use or export to blood
Iron Transport in Blood [14,15,16]	Transferrin (Tf)	Fe^3+^	Binding iron for transport to cells
Cellular Iron Uptake [15,16]	Transferrin Receptors (TfR1, TfR2), Cubilin (kidney)	Fe^3+^ → Cellular Iron	Cellular iron uptake via receptor-mediated endocytosis
Regulation of Iron Homeostasis [15,16]	Hepcidin (liver), Ferroportin degradation	Inhibits Fe^2+^ release by degrading ferroportin	Control of iron concentrations via hepcidin–ferroportin interaction

**Table 2:** Summary of research on sport horses.

Research Area Related to Iron Status	Obtained Results	Publication
Resting serum iron concentrations in sport and recreational horses	Sport horses have higher serum Fe concentrations than recreational horses	Burlikowska et al. (2015) [90]
Effect of different exercise protocols (aerobic of varying intensity and duration) on iron homeostasis	Different types of physical exercise affect iron homeostasis in various ways	Mills et al. (1996) [91]
Iron concentrations as a potential indicator of elimination risk before an endurance ride	Iron concentrations are not reliable indicators for race elimination risk	Bollinger et al. (2023) [92]
Effect of aerobic exercise of varying intensity on plasma ferritin concentrations	Physical exertion elevates plasma ferritin concentrations, with a greater increase observed after prolonged and/or more intense exercise	Hyyppä et al., (2002) [93]
Effect of show jumping on iron homeostasis	Increased serum concentrations of ferritin, iron, and transferrin levels during repeated competition	Assenza et al. (2017) [94]
Effect of show jumping on iron homeostasis	Increased concentrations of iron, ferritin, transferrin, TIBC, and UIBC	Piccione et al. (2017) [95]
Effect of high intensity, short physical exercise (gallop) on iron homeostasis	Significant decrease in serum iron concentration, along with increased TIBC and transferrin saturation after exercise	Abramovitc et al. (2014) [96]
Effect of long-term physical exercise (80-day training program) on iron homeostasis	As the program progressed, transferrin, TIBC, and UIBC concentrations decreased, while ferritin and iron concentrations increased	Assenza et al. (2016) [97]
Effect of high-speed treadmill exercise (5-day protocol) on serum iron concentrations	Serum iron concentration significantly increased during exercise, followed by a gradual decrease	Inoue et al. (2002) [98]

**Table 3:** Iron metabolism monitoring recommendation in horses.

Iron Monitoring Parameter	Significance	Optimal Sampling Time	Recommended Analytical Method
Serum Iron (Fe)	Measures circulating iron concentrations	Baseline, post-exercise (immediate, 30 min, 60 min)	Spectrophotometry
Ferritin	Indicator of iron stores and acute-phase response	Baseline, post-exercise (24 h, long-term)	ELISA
Transferrin	Iron transport protein; key for iron homeostasis	Baseline, post-exercise (immediate, 30 min)	ELISA
Total Iron-Binding Capacity (TIBC)	Measures total iron-binding potential of transferrin	Baseline, post-exercise (immediate, 30 min)	Spectrophotometry
Unsaturated Iron-Binding Capacity (UIBC)	Measures iron-binding capacity available in transferrin	Baseline, post-exercise (immediate, 30 min)	Spectrophotometry
Hemoglobin (Hb)	Essential for oxygen transport	Baseline, post-exercise (immediate, 30 min, long-term)	Hematology analyzer
Red Blood Cell Count (RBC)	Reflects erythropoiesis and overall red cell mass	Baseline and post-exercise (immediate, 30 min, long-term)	Hematology analyzer
Hematocrit (Hct)	Indicates blood volume occupied by red cells	Baseline and post-exercise (immediate, 30 min, long-term)	Hematology analyzer
Hepcidin	Regulates iron absorption and distribution	Baseline, post-exercise (24 h, long-term)	ELISA

**Table 4:** Post-exercise increases in hematocrit (Hct), red blood cell count (RBC), and hemoglobin concentration (Hb) parameters in race horses across different breeds and race distances.

Publication	Hct	RBC	Hb	Breed	Distance
Piccione et al. (2010) [113]	36%	34%	37.5%	Standardbred Horses	1600 m
Piccione et al. (2010) [113]	23.5%	20%	12.3%	Standardbred Horses	2000 m
Wood and Frede (1997) [114]	57.5%	48.1%	46.3%	Quarter Horses and Thoroughbred Horses	from 320 m to 1700 m
Hassan et al. (2015) [115]	no data	45.5%	33.3%	Thoroughbred Horses	1600 m

Presented values were calculated by the authors of the present review based on raw data (pre- and post-race measurements) extracted from the cited publications, and expressed as percentage differences relative to pre-race values. In cases where data for mares and geldings were provided separately, the calculations were performed using the combined results for both groups.

**Table 5:** Suggested diagnostic considerations related to iron status monitoring in sport horses (based on studies [9,109,111,112,113,114,115,116,117,118,119,122] and clinical observations). ↓: low.

**Clinical context**	**Suggested considerations (not Clinical Guidelines)**
↓ Performance/poor recovery	Consider evaluation of Hct, Hb, RBC, serum iron, ferritin, inflammatory markers (SAA, IL-6), and hepcidin (if available). Post-exercise samples (e.g., immediately after exercise and 24 h later) may reveal transient alterations.
Relevant blood tests	CBC (Hct, Hb, RBC), serum iron and ferritin, transferrin saturation (TIBC/UIBC), inflammatory markers (e.g., SAA, IL-6), hepcidin (if available).
Possible indicators of subclinical iron imbalance	Low-normal hematologic values in a fit horse with poor adaptation; history of exertional hemolysis, EGUS, or chronic inflammatory burden; suboptimal recovery after training. Interpretation should be cautious due to physiological variation.
Situations where iron status evaluation may be warranted	Prolonged recovery, post-surgical anemia, chronic blood loss, or suspected iron depletion. Supplementation should be considered only with laboratory confirmation and in the absence of inflammatory confounders.
Potential risks of iron over-supplementation (reported in the literature)	Iron overload (especially with repeated parenteral use), oxidative damage, hepatotoxicity, and in rare cases, acute toxicity. Injectable iron may bypass regulatory barriers; caution is advised.
